# Social Support, Health Behaviors, Self-Esteem, and Successful Aging in a Sub-Saharan African Sample of Older Adults: Test of a Sequential Mediation Model

**DOI:** 10.1093/geroni/igae030

**Published:** 2024-03-06

**Authors:** Chima Charles Igbokwe, Blessing Nneka Ome, Runcie C W Chidebe, Beatrice Chinwe Igbokwe, Mary Basil Nwoke, Chidiebere Wisdom Obioha, Benard Chibuike Okechi, JohnBosco Chika Chukwuorji

**Affiliations:** Department of Human Kinetics and Health Education, University of Nigeria, Nsukka, Enugu, Nigeria; Department of Psychology, University of Nigeria, Nsukka, Enugu, Nigeria; Department of Sociology and Gerontology, Miami University, Ohio, USA; Scripps Gerontology Center, Oxford, Ohio, USA; Project PINK BLUE – Health and Psychological Trust Centre, Abuja, Nigeria; Department of Psychology, University of Nigeria, Nsukka, Enugu, Nigeria; Department of Psychology, University of Nigeria, Nsukka, Enugu, Nigeria; Department of Psychology, University of Nigeria, Nsukka, Enugu, Nigeria; Department of Psychology, University of Nigeria, Nsukka, Enugu, Nigeria; Department of Psychology, University of Nigeria, Nsukka, Enugu, Nigeria; Department of Psychology, Nile University of Nigeria, Abuja, Nigeria

**Keywords:** Health behaviors, Self-esteem, Social support, Sub-Saharan Africa, Successful aging

## Abstract

**Background and Objectives:**

Previous research demonstrates that social support facilitates successful aging across all cultures. However, the factors that potentially mediate the link between social support and successful aging remain unclear. This study examined whether a healthy lifestyle and self-esteem mediate the association between social support and successful aging. It was hypothesized that the relationship between social support (family, friends, and significant other) and successful aging would be serially mediated by both healthy lifestyle and self-esteem.

**Research Design and Methods:**

Participants were 479 Nigerian retirees (53.4% female) aged 60 to 90 years (*M*_age_ = 64.81, *SD* = 6.86). They provided information on relevant demographic variables and completed the following measures: Fantastic Lifestyle Checklist (Fitness Appraisal), Rosenberg Self-esteem Scale, Multidimensional Scale of Perceived Social Support Scale, and Successful Aging Inventory. Three separate regression models (family, friends, and significant other dimensions of social support) were conducted using the Hayes PROCESS macro for SPSS with 5,000 bootstrap estimates.

**Results:**

Controlling for age and sex, family support, significant other support, friends support, healthy lifestyle, and self-esteem were directly associated with successful aging. The association between family support and successful aging was mediated by healthy lifestyle; and this was also seen for friends’ support and significant other support. The sequential path from social support to successful aging through healthy lifestyle, and then via improved self-esteem, was significant for family support and significant other support, but not friends support.

**Discussion and Implications:**

Findings suggest that middle-aged to older adults who have strong support from their families and significant others may be more likely to engage in healthy behaviors and, in turn, experience higher levels of self-esteem, thereby aging well.

**Translational Significance:** There is a lack of research on successful aging and its mediating links in Sub-Saharan Africa (SSA). Our findings underscore the importance of family and significant other support in improving healthy lifestyles and self-esteem as pathways to successful aging. Although successful aging requires individual efforts to adopt a healthy lifestyle in Western countries, we argue that family support and systems are crucial in SSA. Our findings challenge Nigeria’s strategic aging roadmap and the African Union’s plan of action on aging to strengthen family systems while exemplifying the uniqueness of the SSA in global aging studies.

## Background

Old age is desirable and inevitable, but it is considered a stressful life stage in many parts of Sub-Saharan Africa (SSA) due to limited aging policies, lack of social security, and scanty old age benefits (Adebowale et al., 2022). As part of efforts to explore the psychology of aging beyond disease and disability and interrogate doing well in later life, successful aging emerged. Successful aging refers to a situation where an individual is in a state of low probability of diseases and disability, has good mental and physical functioning, and has active engagement with life (Rowe & Kahn, 1997). Several multidisciplinary seminal studies have been conducted on successful aging, and there is incontrovertible evidence that older adults who are aging successfully reduced the risk of mortality by 50% compared to those who were not (Mao et al., 2022). This underscores the importance of successful aging in gerontology/geriatrics.

Despite substantial interest in successful aging in Western cultures, relatively little research has explored this concept in Africa and SSA. This is surprising for several reasons. First, the African continent will experience an increase in the number of older adults from 74.4 million in 2020 to 235.1 million by 2050 (He et al., 2020). Second, older adults in Africa occupy critical leadership roles in governance, political and economic activities, family, community, and other social institutions. At the same time, policies are not in place to support these members of society. Universal health coverage for this population is not widely available, the existing health system is weak, and long-term care services and support are underdeveloped (Tumusiime et al., 2019). Taken together, it is important to examine what successful aging “looks like” in this cultural context and to explore how biopsychosocial factors such as social support, health behavior, and self-esteem influence successful aging in this population.

## Social Support and Successful Aging

Existing literature has shown that social support is associated with successful aging at different levels. For example, Vaillant and Mukamal (2001) followed two cohorts for 60 years and they found that social support could influence successful aging in later years. Howie et al. (2014) conducted a cross-sectional descriptive study of 154 older adults residing in an assisted living community using the Successful Aging Inventory and the Lubben Social Network Scale. They found a moderate correlation between social support and successful aging. Other studies (e.g., Almeida et al., 2009; Sharma, 2020) found that older adults who had regular contact with friends and family were more functionally active than those who did not. They concluded that social support (including support from family and friends) could improve physical health and psychological well-being by providing emotional, informational, and tangible assets, which can help middle-aged to older adults cope with stress and reduce the adverse impact of aging-related changes. In Nigeria, Chukwuorji et al.’s (2017) study of the Biafra War generation also reported that family support was moderately and positively associated with successful aging. More importantly, several recent systematic reviews and meta-analyses showed convergent evidence of the positive association of social support as a health asset in old age (Abud et al., 2022; Lee et al., 2022).

Given that social support is a multifaceted phenomenon that is influenced by a person’s sociopolitical environment, socialization process, and personal values, among other things (Dambi et al., 2018; Pérez-Villalobos et al., 2021), it is relevant to differentiate the perceived support from family, friends, and significant others with successful aging. The nature of social support in terms of those who provide the support (i.e., source) can come from family, friends, and significant other (Zimet et al., 1988), but several studies have utilized either one aspect of support or conceptualized the construct as unidimensional. Family support assists older adults in gaining access to a wide range of services and supports, as well as a network of community services that support older adults. In a similar manner, support from friends and significant others (e.g., doctors, colleagues, and so on) provides a feeling of shared affection, mutual respect, personal worth, and care (Banovcinova & Baskova, 2016). As noted in literature among younger populations (Hatteberg, 2021), studies that fail to consider the sources of support may lose important information because one specific dimension may be a stronger predictor of successful aging. In their efforts to clarify the mechanisms linking social support to favorable health outcomes, some models of the stress-buffering role of social support also suggest that the efficacy of support should be predicated, in part, on the source of such support (Cohen & McKay, 1984; Thoits, 1986, 2011). In the present study, we will give attention to the three dimensions of social support to provide a more nuanced understanding of the unique patterns of association that may hold for these dimensions with successful aging.

## Health-Related Behavior, Self-Esteem, and Successful Aging

Although social support is associated with successful aging, health behavior is also critical in this aging process, and certain behaviors may contribute to successful aging (Amini et al., 2021; Chen et al., 2021). Studies among Indonesians (Oktaviani et al., 2022), Puerto Ricans (Lee-Bravatti et al., 2021), and Norwegians (Bosnes et al., 2019) reported that health-related behaviors and changes such as quitting smoking, performing medium physical activity, and increasing protein intake were protective factors for successful aging. For the most part, an integrated healthy lifestyle is related to a better chance of overall successful aging (Ulfa & Sartika, 2019). Guided by psychosocial models of successful aging (Bowling & Dieppe, 2005), personal resources (e.g., self-esteem) have been observed to be a crucial factor in the aging process (Abud et al., 2022). Self-esteem, which is the correspondence between the ideal and actual self-concept of an individual (Gómez-Ortiz et al., 2019), is characterized by confidence, high levels of belief, and satisfaction with oneself (Baumeister & Vohs, 2018). Among older adults in Australia (Chochovski et al., 2016), Iran (Franak et al., 2015), Croatia (Pluzarić et al., 2016), and China (Sun et al., 2017), self-esteem was associated with increased odds of aging successfully.

## Social Support, Health-Related Behavior, Self-Esteem, and Successful Aging

The mechanisms that may explain the association of social support on health and longevity have been highlighted as one of the research areas that will provide a more nuanced understanding of how social factors influence well-being outcomes across the lifespan (Vila, 2021). However, only a few studies have investigated health behavior and self-esteem as potential mediators of the link between social support and successful aging. For example, Wang & Geng (2019) found that lifestyle mediated the relationship between socioeconomic status and health, whereas Poortinga (2006) found no mediation relationship with lifestyle factors for English participants. In the Netherlands, physical activity, but not nutrition and sleeping, has been identified as a mediating factor in the relationship between social capital and individual health (Mohnen et al., 2012). A study based on a Finnish health survey observed that part of the association between social participation, networks, and health was explained by physical activity (Nieminen et al., 2013), a health-promoting behavior. Thanakwang Soonthorndhada (2011) found that health-promoting behaviors had a powerful influence on healthy aging and played a significant role in mediating the relationship between family and friendship support and healthy aging among Thai community-dwelling older adults. Xue and Cheng (2017) asserted that lifestyle factors such as a healthy diet and physical activity mediated the relationship between social capital (social trust and social relationship) and health (self-rated health and psychological well-being). Zhang et al. (2020) reported that a healthy lifestyle is a mediator of the relationship between self-perception of aging and mortality in middle-aged and older adults.

Self-esteem as a mediator between intergenerational support and well-being in old adults has been verified in previous studies (Tian, 2016; Yang et al., 2022).

## Theoretical Framework

We adopted King’s (1981) theory of open systems and model of human interaction to support the connections of the source and function of social support with self-esteem, health-related behavior, and the outcome of successful aging. Based on King’s model, health and well-being is determined by social functioning in the context of three interacting systems, namely, personal, interpersonal, and social systems (King, 1981). The personal system emphasizes one’s perceptions of self, time, and space within the context of the other two systems. The interpersonal system refers to “two, three, or more individuals interacting in a given situation” (King, 1981, p. 54); whereas the social system consists of “units of analysis in a society in which individuals form groups to carry on activities of daily living to maintain life, health and happiness” (King, 1981, p. 115). This theoretical framework of three interacting social systems with permeable boundaries is consistent with extant theoretical models of social support (Kaplan, 1985). There is a shared assumption that social support originates from a source to a person (system), is accessible to a person (within a system), and may be transmitted from one person (system) to another. As middle-aged to older adults in SSA retire, they receive social support from different sources such as the community, friends, significant others, and family. In this transmission process, the support system influences the activities the person engages in and provides the individual with a sense of self, which in turn affects the attainment of successful aging.

## The Present Study

**Figure 1.**
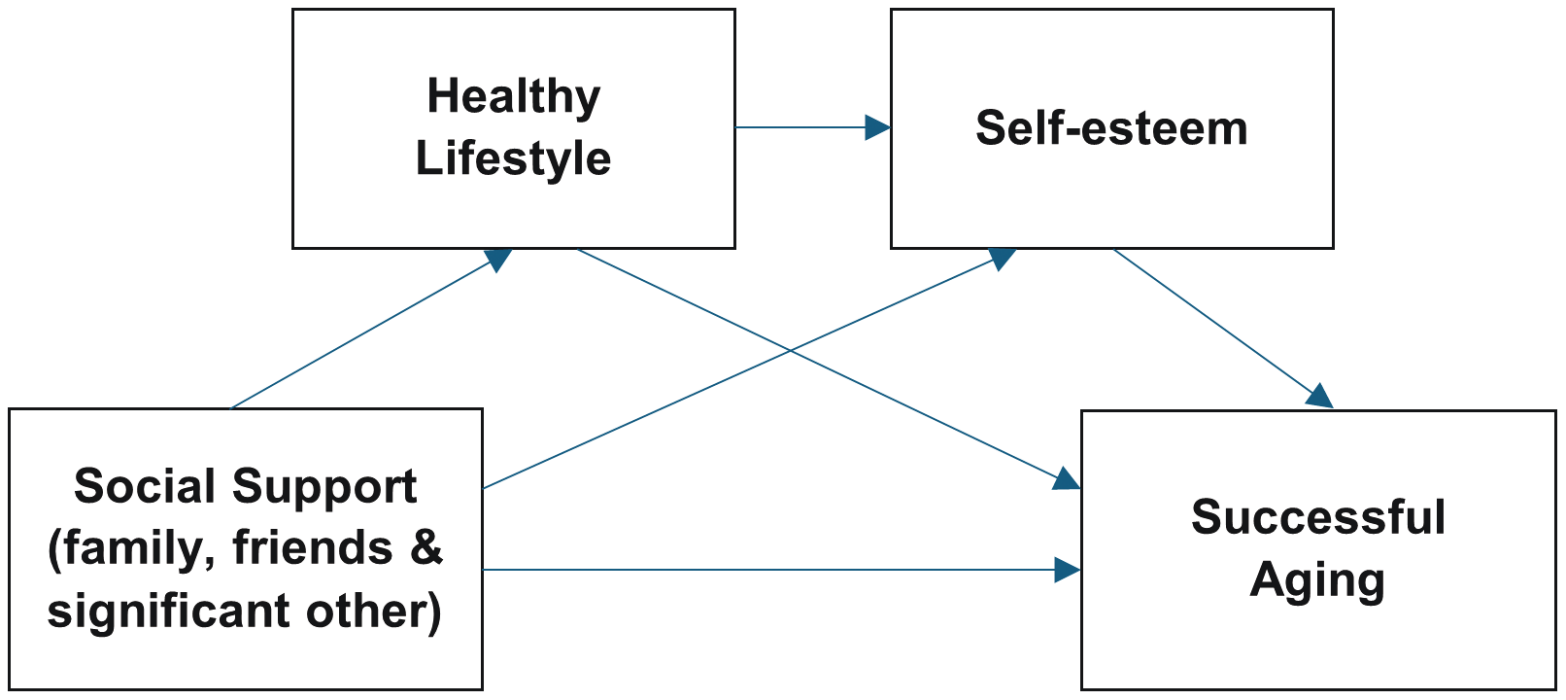
Conceptual model of social support, health behavior, self-esteem, and successful aging.

Evidence has also shown that health-related behavior mediated the link between social resources and health outcomes (Mohnen et al., 2012; Nieminen et al., 2013; Xue & Cheng, 2017; Zhang et al., 2020). Health-promoting behaviors are a predictor of self-esteem in old adulthood (Chen et al., 2022; Chia et al., 2023; Haera & Panyachai, 2023; Rababa et al., 2021). Furthermore, the pathway between social support and health in old age has been observed to have multiple mediators. For instance, in Yang et al.’s (2022) study, both loneliness and self-esteem serially mediated the link between social support and self-esteem. Specifically, intergenerational emotional support ameliorates loneliness, which in turn improves self-esteem and thereby leads to increased well-being. Chen et al. (2022) also obtained evidence of the sequential mediating role of a sense of meaning in life and self-esteem in the association between physical exercise and subjective well-being in older adults. Based on the literature, it is plausible that healthy lifestyles and self-esteem may be mediating variables between social support and successful aging. Otherwise, older adults with high social support are more likely to engage in healthy lifestyles, which enhance their self-esteem and thereby contribute to aging successfully (see Figure 1). The order of the serial mediation pathway was based on existing empirical work and not with much theoretical basis. The theoretical framework represents aspects of direct pathways of relationships between the predictors and successful aging but does not completely characterize the mediation hypotheses.

The objectives of the present study are to utilize SSA data to examine whether: (1) social support, healthy lifestyle, and self-esteem are related to successful aging and (2) healthy lifestyle and self-esteem mediate the relationship between social support and successful aging. Our study further seeks to add to the existing literature by recognizing the multidimensionality of the social support concept and measurement, which has been emphasized as a missing element of most studies on social support and successful aging (see Vila, 2021). We hypothesize that:

(1) A higher healthy lifestyle will be associated with greater successful aging.
(2) Support from family, significant others, and friends support will be positively associated with successful aging.
(3) Higher self-esteem will be associated with greater successful aging.
(4) The association between social support (family, significant others, and friends) and successful aging will be mediated by a healthy lifestyle.
(5) Both healthy lifestyle and self-esteem will sequentially mediate the association between social support and successful aging.

## Method

### Sample and Procedure

Participants in this study were 479 retirees (*M*_age_ = 64.81 years; 53.4% female) who were recruited during the September and October 2019 monthly verification exercise at the Enugu State Pension Board (ENSPB). The ENSPB is a government agency based in Enugu state—a mainland state in the South-East region of Nigeria and the political capital of the Igbo Nation. Nigeria is the most populous country in SSA. The retirees were addressed by one of the researchers and invited to voluntarily participate in the study. Only retirees who signed the informed consent forms were recruited for the study. Given that an average level of English language literacy is required for employment in the public service, we administered the questionnaires in English. Ethical clearance for the study was obtained from the Psychology Research Ethics Committee of the University of Nigeria, Nsukka, and permission to conduct the study was given by the Director of ENSPB.

### Instruments

The following measures were utilized for data collection: the fitness appraisal subscale of the Fantastic Lifestyle Checklist (FA-FLC; Wilson, 1984), the Rosenburg Self-esteem Scale (RSES; Rosenberg, 1965), the Multidimensional Scale of Perceived Social Support (MSPSS; Zimet et al., 1988), and the Successful Aging Inventory (SAI; Troutman et al., 2011).

The FA-FLC is a 25-item questionnaire that measures lifestyle factors (physical, psychological, and social aspects) measured on a 5-point Likert scale ranging from 1 *(Almost never)* to 5 *(Almost always)*. Sample items include *I use seatbelts, I am a positive, or optimistic thinker*. Wilson (1984) reported solid psychometric properties (Cronbach’s α = 0.69; intraclass correlation coefficient = 0.92; Kappa index [construct validity] = 0.58). The scale was used in a previous study in Nigeria (Shehu et al., 2009). For this present study, Cronbach’s α = 0.79.

For each of the 10 RSES items, participants indicate on a 4-point Likert scale ranging from 1 *(strongly disagree)* to 4 *(strongly agree)* the extent to which they agree with each statement. Sample items include: *On the whole I am satisfied with myself, At times I think I am no good at all*. After reverse scoring the negatively worded items, all items are summed to yield a total score and higher scores indicate better health-promoting behaviors. Rosenberg (1965) reported α of 0.72, whereas Ifeagwazi (2010) and Ifeagwazi and Ezema (2010) reported good α coefficients in Nigerian samples. Cronbach α for the current study (α = 0.77) was acceptable.

The MSPSS is a 12-item scale that measures perceived support from family, friends, and significant others. Each item is rated on a scale ranging from 1 *(very strongly disagree)* to 7 *(very strongly agree)*. Sample items include: *There is a significant other who is around when I am in need; My family really tries to help me; I can talk about my problems with my friends.*Zimet et al. (1988) reported α of 0.82, whereas other studies in Nigeria reported good psychometric indices (e.g., Chukwuorji et al., 2020; Ifeagwazi & Chukwuorji, 2014). For the present study, the overall α was 0.92; subscale α were 0.86 (Family), 0.80 (Friends), and 0.84 (Significant Others). The items for each of the dimensions were summed to obtain the score for the particular subscale with higher scores indicating high perception of support on the scale.

The 20-item self-report SAI measures how people feel about their aging processes on a 5-point scale ranging from 1 *(Hardly ever)* to 5 *(Almost always)*. Sample items include: *I look forward to the future; I am in a positive, pleasant mood; and I feel that I serve a purpose in this world*. The α was 0.86 (Troutman et al., 2011). Among Nigerian older adults, Chukwuorji et al. (2017) reported α of 0.88. In the current study, the α was 0.87. To obtain a total score for successful aging, the sum of all items was calculated, and higher scores reflect higher successful aging.

### Statistical Analysis

We adopted the complete case (or available case) analysis or listwise deletion of missing data by simply omitting those cases with the missing data and analyzing the remaining data. Tests of normality using skewness and kurtosis indicated that the data was normally distributed. Pearson’s correlation was used to establish the relationships between the relevant demographic variables and the major variables. The PROCESS Macro for SPSS (Hayes, 2018) was employed to estimate the path coefficients in the mediator model. The macro produces direct total association values, indirect association estimates for diverse intermediate variables, standard errors, and bias-corrected bootstrapped confidence intervals (*CI*s) obtained from the sample distribution. Bootstrapping is a resampling technique that estimates population variables by continuously resampling an empirical sample (Ong, 2014). The merits of bootstrapping over the traditional *p* values in tests of hypotheses have been highlighted in the existing literature (e.g., Cumming, 2014). For the present analyses, we used Model 6 of the PROCESS, which permits us to add two mediators (healthy lifestyle and self-esteem). The three dimensions of social support were the predictors, while successful aging was the outcome variable. The mediation (indirect) association is supported when zero does not occur between the upper and lower limits of the *CI* (Fritz & MacKinnon, 2007). The PROCESS macro is widely used in tests of mediation hypotheses because of its higher power and greater accuracy compared to Sobel’s test (e.g., Eze et al., 2020; Spalding et al., 2019).

## Results

Participants were mostly married (84.6%); 6.9% were widows/widowers, 5.8% were separated, and 2.7% were never married. With respect to ethnicity, participants were overwhelmingly Igbo (99.6%) and Christian (99.4%). More than three-quarters (76.0%) resided in an urban area. The majority of the participants indicated that they had tertiary education (88.9%; see Table 1).

**Table 1.** Demographic Characteristics of Study Participants (*N* = 479)

Characteristic	*M* (*SD*)	Range	*N* (%)
Age (years)	64.81 (6.86)	60–90	
Sex			
Male			223 (46.6)
Female			256 (53.4)
Marital status			
Married			405 (84.6)
Single			13 (2.7)
Separated/divorced			28 (5.8)
Widowed			33 (6.9)
Education			
Primary education			6 (1.3)
Secondary education			47 (9.8)
Tertiary			426 (88.9)
Occupation			
Retired			471 (98.3)
Skilled manual worker/farmer			4 (0.8)
Professional			4 (0.8)
Religion			
Christian			476 (99.4)
Muslim			1 (0.2)
Others			2 (0.4)
Ethnic group			
Igbo			477 (99.6)
Others			2 (0.4)
Locality			
Urban			373 (76.0)
Rural			115 (24.0)
Family support	18.49 (5.92)	4–28	
Friends support	17.76 (4.97)	4–28	
Significant other	17.51 (6.37)	4–28	
Lifestyle	81.03 (14.58)	37–124	
Self-esteem	23.01 (4.35)	11–40	
Successive aging	64.30 (12.78)	28–100	

*Note*: *SD* = standard deviation.

We conducted an independent sample *t* test to examine sex differences in social support, lifestyle, self-esteem, and successful aging. For the male participants, there were statistical associations between significant other support *t*(477) = 2.59, *p* < .01, family support *t*(477) = 3.11, *p* < .01, and friend support *t*(477) = 1.99, *p* < .05, compared to female participants. Whereas women (*M *= 18.81, *SD* = 6.32) had higher scores than men (*M = *18.31, *SD *= 6.34) on significant other support. On family support, men (*M* = 19.38, *SD *= 5.77) had higher scores than women (*M* = 17.71, *SD* = 5.95), and on friend support, men (*M *= 18.24, *SD* = 4.72) also had higher scores than women (*M *= 17.34, *SD* = 5.15). There were statistical associations between healthy lifestyle *t*(477) = 3.63, *p* < .001, self-esteem *t*(477) = 2.04, *p* < .05, and successful aging *t*(477) = 3.70, *p* < .001, in men compared to women. For men (*M *= 83.62, *SD* = 15.53), they had higher scores than women (*M* = 78.78, *SD* = 13.32) on healthy lifestyles. For self-esteem, men (*M *= 23.44, *SD* = 4.20) had higher scores than women (*M* = 22.64, *SD* = 4.46), as well as in successful aging (men: *M* = 66.59, *SD *= 4.72; women: *M* = 62.32, *SD *= 12.86).

Table 2 presents the correlations between the demographic, predictor, mediation, and outcome variables. As can be seen in the table, education was not significantly related to any of the major variables in the study. Age was also positively correlated with healthy lifestyles. Age and sex were controlled for in the subsequent regression analysis due to their significant association with one or more major variables. Higher support from significant others, family, and friends, higher healthy lifestyles, and higher self-esteem were all associated with higher successful aging with correlation coefficients ranging from low to moderate.

**Table 2.** Correlations of Demographic Variables, Social Support (Family, Friends, and Significant Other), Healthy Lifestyle, Self-Esteem, and Successive Aging

Variables		1	2	3	4	5	6	7
1	Age	—						
2	Education^a^	−0.02	—					
3	Significant other	0.04	−0.04	—				
4	Family support	0.06	0.00	0.70***	—			
5	Friends support	0.07	−0.08	0.61***	0.61***	—		
6	Healthy lifestyle	0.20***	0.05	0.42***	0.46***	0.31***	—	
7	Self-esteem	0.08	−0.04	0.18***	0.19***	0.26***	0.28***	—
8	Successive aging	0.02	−0.06	0.45***	0.46***	0.39***	0.48***	0.26***

*Notes*: ^a^Education was coded 0 = primary education, 1 = secondary education, 2 = tertiary education.

***p < .001.

Table 3 presents the results of the analysis of direct association between the variables, which was divided into three model summaries. Model summary 1 shows the direct association of family support, healthy lifestyle, and self-esteem when healthy lifestyle and self-esteem were added as mediators. Model summary 2 shows the direct association of support from significant others, healthy lifestyle, and self-esteem when healthy lifestyle and self-esteem were added as mediators. Model summary 3 shows the direct association of friends’ support, healthy lifestyle, and self-esteem when healthy lifestyle and self-esteem were added as mediators. Age and sex were controlled for in all models due to their significant relationships with the main variables in the study (see Table 2).

**Table 3.** Analysis of Direct Association Between Age, Sex, Family Support, Healthy Lifestyle, and Self-Esteem

Variable	*B*	β	*t*	*p* Value	95% CI	*R* ^2^	*F*
A							
Age	−0.15	−0.08	−2.10	.036	[−0.30, −0.10]	0.32	44.80 (5, 472)***
Sex	−2.06	−0.08	−2.50	.041	[−4.02, −0.09]		
Family support	0.61	0.28	6.52	.000	[0.42, 0.79]		
Healthy lifestyle	0.28	0.32	7.17	.000	[0.20, 0.36]		
Self-esteem	0.33	0.11	2.87	.004	[0.11, 0.56]		
B							
Age	−0.15	−0.08	−2.03	.043	[−0.29, 0.01]	0.57	45.88 (5, 473)***
Sex	−2.14	−0.08	−2.15	.032	[−4.10, −0.19]		
Significant other	0.57	0.29	6.80	.000	[0.41, 0.74]		
Healthy lifestyle	0.29	0.33	7.53	.000	[0.21, 0.36]		
Self-esteem	0.33	0.11	2.83	.005	[0.10, 0.56]		
C							
Age	−0.18	−0.10	−2.42	.016	[−0.31, −0.03]	0.56	43.41 (5, 473)***
Sex	−2.35	−0.09	−2.34	.020	[−4.32, −0.38]		
Friends support	0.64	0.25	6.09	.000	[0.43, 0.85]		
Healthy lifestyle	0.33	0.38	9.04	.000	[0.26, 0.41]		
Self-esteem	0.25	0.09	2.13	.034	[0.02, 0.49]		

*Notes*: CI = confidence interval.

***p < .001.

The results in model summary 1 showed that family support was positively associated with successful aging (β = 0.28, *p* < .001). Each unit increase in family support was associated with a 0.61 unit increase in successful aging. Healthy lifestyle was positively associated with successful aging (β = 0.32, *p* < .001); each unit increase in healthy lifestyle was associated with a 0.28 unit increase in successful aging. Self-esteem was positively associated with successful aging (β = 0.11, *p* < .01); each unit increase in self-esteem was associated with a 0.33 increase in successful aging.

The results in model summary 2 showed that support from significant others was positively associated with successful aging (β = 0.29, *p* < .001). Each unit increase in support from significant others was associated with a 0.57 increase in successful aging. Healthy lifestyle was positively associated with successful aging (β = 0.33, *p* < .001). Each unit increase in healthy lifestyle was associated with a 0.29 increase in successful aging. Self-esteem was positively associated with successful aging (β = 0.11, *p* < .01). Each unit increase in self-esteem was associated with a 0.33 increase in successful aging.

The results in model summary 3 showed that friends’ support was positively associated with successful aging (β = 0.25, *p* < .001). Each unit increase in friends’ support was associated with a 0.64 increase in successful aging. Healthy lifestyle was positively associated with successful aging (β = 0.38, *p* < .001). Each unit increase in healthy lifestyle was associated with a 0.33 increase in successful aging. Self-esteem was positively associated with successful aging (β = 0.09, *p* < .05). Each unit increase in self-esteem was associated with a 0.25 increase in successful aging.

The direct association between social support and successful aging is established in Table 3. The regression coefficients were all significant and positive for all its dimensions, which indicates that higher social support from family, significant others, and friends was associated with increased successful aging. Social support from friends emerged as the most robust predictor of successful aging and was closely followed by family support. Support from significant others was next with a slightly lesser regression coefficient.

Table 4 presents the results of the multiple mediation analysis. A healthy lifestyle mediated the association of family support on successful aging. Self-esteem did not mediate the association of family support on successful aging, but its indirect association became significant when healthy lifestyle was the first mediator (family support, healthy lifestyle, self-esteem, successful aging). A healthy lifestyle mediated the association of support from significant others on successful aging. Self-esteem did not mediate the association of support from significant others on successful aging, but its indirect association became significant when healthy lifestyle was the first mediator (significant others, healthy lifestyle, self-esteem, successful aging). A healthy lifestyle mediated the association of friends’ support on successful aging. Self-esteem did not mediate the association of support from significant others on successful aging. Neither healthy lifestyle nor self-esteem mediated the association of friend support on successful aging when healthy lifestyle was the first mediator and self-esteem was the second mediator (i.e., friends support, healthy lifestyle, self-esteem, successful aging).

**Table 4.** Completely Standardized Bootstrap Tests of Mediating Association of Family Support, Significant Other, and Friends Support

Variables	*B*	95% CI
Family support		
FS → HLS → SA	0.14	[**0.10, 0.19**]
FS → SE → SA	0.01	[−0.00, 0.03]
FS → HLS → SE → SA	0.01	[**0.01, 0.03**]
Significant other		
SP → HLS → SA	0.13	[**0.09, 0.18**]
SP → SE → SA	0.09	[−0.00, 0.03]
SP → HLS → SE → SA	0.01	[**0.01, 0.03**]
Friends support		
FS^*^ → HLS → SA	0.11	[**0.07, 0.15**]
FS^*^ → SE → SA	0.02	[−0.00, 0.04]
FS^*^ → HLS → SE → SA	0.01	[0.00, 0.01]

*Note*: CI = confidence interval; FS = family support; FS* = friends support; HLS = healthy lifestyle; SA = successful aging; SE = self-esteem; SP = significant other. Bold fonts are significant mediation paths.

## Discussion

Despite long-standing social support and successful aging in gerontological literature, relatively little research has been explored in SSA. Further, little if any research has explicitly focused on factors that potentially mediate the link between social support and successful aging. The goal of the present study was to examine whether a healthy lifestyle and self-esteem are mediators of how social support positively influences successful aging. It was hypothesized that the relationship between social support (from family, friends, and a significant other) and successful aging would be serially mediated by both healthy lifestyle and self-esteem. The main findings show that support from family, significant others, and friends, healthy lifestyle, and self-esteem were directly associated with successful aging. In addition, the association between family support and successful aging was mediated by healthy lifestyle, support from friends, and a significant other. Although the sequential path to successful aging through healthy lifestyle and improved self-esteem was significant for family support and significant other support; friends’ support was not significant.

### Healthy Lifestyle Mediates the Association Between Family Support and Successful Aging in SSA

The finding that family support and successful aging were mediated by a healthy lifestyle is consistent with a previous study (e.g., Chukwuorji et al., 2020). Specifically, participants who engaged in physical activities and ate a healthy diet (i.e., well-balanced and including vegetables), limited their alcohol intake and did not smoke were more likely to report more family support, which together influenced their potential of aging successfully. Based on the findings of this present study, we speculate that a higher lifestyle is associated with greater successful aging in an SSA sample. This finding provides some insight into the intersection of family support, successful aging, and healthy lifestyle in SSA. From Bowen’s (1966) family systems theoretical standpoint, the interconnections in the family systems are interlocking forces that shape family functioning and individual behaviors such as healthy lifestyles (Brown, 1999).

Literature abounds that family members with unhealthy lifestyles influence others (Johannsen et al., 2006; Savage et al., 2007) and that family members assume social control agents in shaping the healthy/unhealthy lifestyles of older adults (Tucker, 2002). With this understanding, a healthy lifestyle is interconnected with family support and the system. Much of the studies were from Western societies, and so our findings extend findings from other cultural contexts to the African context. One implication of this finding is that, despite the assertions by Rowe and Kahn (1998) that aging successfully requires individual efforts in adopting healthy lifestyles, choices, and behaviors, our finding points to the role of family support in successful aging in SSA. For example, an older adult who desires to age successfully could utilize Bowen’s “differentiation of self” and make an individual choice of adopting a healthy lifestyle, which may be supported by the family or not. As middle-aged to older adults and SSA communities make efforts toward improving aging and attaining successful aging; the mediating role of a healthy lifestyle should be considered in that effort or intervention.

### Support From Family, Significant Others, and Friends, Healthy Lifestyles, and Self-Esteem Are Directly Associated With Successful Aging

Interestingly, support from family, significant others, and friends, maintaining a healthy lifestyle, and self-esteem were directly associated with successful aging. One explanation for this finding is that most SSA countries such as Nigeria are still relatively traditional societies where informal support networks influence the aging process (Ola, 2015). In many of the SSA communities, the leadership of clans and social groups is still bestowed on older adults based on their chronological age. Older adults have been traditionally viewed positively as repositories of wisdom and knowledge (Cohen & Menken, 2006), contributing to their self-esteem. Arguably, the above cultural values in SSA manifest in this association between self-esteem and successful aging. At the same time, social security programs and optimum pension and retirement systems are still lacking in many countries in the SSA (Bailey & Turner, 2002); in countries where they exist, only older adults working in some formal economy or public settings benefit from such programs. As such, this could help explain the importance of informal sources of support while making important aging decisions.

### Sequential Path From Social Support to Successful Aging Through Healthy Lifestyle and Self-Esteem is Significant for Family Support

Interestingly, the sequential path from social support to successful aging through healthy lifestyles, and then via improved self-esteem was significant for family support and significant other support, but not significant for friends’ support. This finding is consistent with those found in Chang et al. (2022) and Vaillant and Mukamal (2001). This explains the type of social network and the importance of support to the participants in the aging process. For instance, family support to older adults is often seen as an honor, and family members’ responsibility to maintain family connections and relationships (Adamek et al., 2020). This is enshrined in the family system, where members of the families are expected by the culture to support and care for older adults’ welfare including paying for their healthcare needs, housing, and other social needs when health insurance and pension are unavailable. Hence, seeking or asking for support from family and significant others is culturally easy and may not affect self-esteem. On the other hand, friends and their support are viewed as exogenous from the individual, unlike the family and significant others who are more endogenous to the individual. Our finding is further supported by Bowen’s (1966) family systems theory, which explains how members of families are intensely connected emotionally and the role of “emotional fusion.”

### Limitations of the Study and Suggestions for Further Studies

The design of this study is cross-sectional, and it has all the attendant shortcomings of cross-sectional research, for example, common-method bias. The participants were retirees who had some level of education. Thus, caution is needed in generalizing the findings to all middle-aged to older adults in Nigeria, SSA, and Africa. In terms of religion and ethnicity, they were mostly Christians and Igbos. About half of the Nigerian population—49.6% (World Population Review, 2023) are Muslims and Igbos are the third largest ethnic group in Nigeria, they are just 18% (World Atlas, 2019). It is unclear whether the findings can apply to middle-aged to older adults in other ethnocultural groups within the country and across SSA. Besides, without a longitudinal design, it is difficult to determine whether successful aging as the study’s outcome followed the predictors or the predictors resulted from the outcome. Longitudinal research is important to determine the potential mechanisms and the dynamics of the relationships we found in this study over time. Perhaps, successful aging may be both an influence of and an outcome of social support; and self-esteem could lead to better social support and a healthy lifestyle thereby producing a different outcome for successful aging. In sum, the associations we found in this study cannot be said to be causal associations. Future studies should recognize these limitations and possibly add other variables including depression and cognitive functioning.

## Conclusion

This study provided a new explanation of healthy lifestyle and self-esteem as mediators between social support and successful aging in an understudied population in SSA. Using a cross-sectional design, the findings demonstrated that middle-aged to older adults who have strong support from their families and significant others may be more likely to engage in healthy behaviors and, in turn, experience higher levels of self-esteem, thereby aging more successfully. As the African Union continues to promote the revised Policy Framework and Plan of Action on Aging 2022, we hope that African Heads of State and Governments consider our findings on the intersection of healthy lifestyle, family system, and successful aging. As this study contributes to the continental call on closing the gaps in aging research and homegrown gerontology/geriatrics, we believe that our findings will be useful reference material for the implementation of the Nigeria National Senior Citizens Centre’s 2022–2023 strategic roadmap on aging.
